# Characteristics and Outcomes of Locally Recurrent Retroperitoneal Sarcoma After First Relapse in a Single Tertiary Asian Centre and Applicability of the *Sarculator*

**DOI:** 10.3389/fonc.2021.730292

**Published:** 2021-11-25

**Authors:** Hui Jun Lim, Ruxin Wong, Yen Sin Koh, Zhirui Shaun Ho, Chin-Ann Johnny Ong, Mohamad Farid, Ching Ching Melissa Teo

**Affiliations:** ^1^ Department of Sarcoma, Peritoneal and Rare Tumours (SPRinT), Division of Surgery and Surgical Oncology, National Cancer Centre Singapore, Singapore, Singapore; ^2^ Department of Radiation Oncology, National Cancer Centre Singapore, Singapore, Singapore; ^3^ Department of Medical Oncology, National Cancer Centre Singapore, Singapore, Singapore

**Keywords:** retroperitoneal sarcoma, recurrence, validation, nomogram, *Sarculator*

## Abstract

**Objective:**

Retroperitoneal sarcomas (RPS) comprise of 15% of soft tissue sarcomas where five-year overall survival rate is about 50%. Locoregional recurrences are observed in up to 50% of patients within the first five years following resection. Various factors have been shown to influence survival outcomes, such as histological subtype and tumour size. A nomogram for first relapse locally recurrent RPS was developed using 602 patients from 22 centres. The recurrent RPS *Sarculator* is available in an electronic interface and includes variables of age, size, margins of re-resection, radiotherapy, chemotherapy and histology to predict for 6-year disease-free survival (DFS) and overall survival (OS). It has not been validated externally. This study aims to validate the *Sarculator* recurrence nomogram in predicting the survival outcomes of recurrent RPS in an Asian population as well as examine relapse patterns.

**Methods:**

Patients diagnosed with first recurrent RPS from 1 January 2000 to 31 December 2017 with first local relapse and eligible for curative re-resection were retrospectively analysed. The type of surgery was unique for individual patients and suggestions of adjuvant therapy were based on globally recognised standards. Patients were followed up every 3 to 4 months post-operatively for the first 2 to 3 years and 6-monthly to a year thereafter. A R0 or R1 margin is deemed as complete resection, including a microscopically negative margin (R0) and microscopically positive but macroscopically clear margin (R1). R2 is classified as an incomplete resection with tumour rupture or remaining disease. Harrell’s C concordance index was used to determine the nomogram’s discriminative ability and calibration plots were used to assess accuracy. For the calibration, the patients were divided into 3 groups. Death data was retrieved from the National Birth and Death registry for accuracy.

**Results:**

There were 53 patients included in this study. Patient and tumour characteristics have been summarised in Table 1. All patients had their second resection at a single centre. 66.0% had their first resection at the same centre. The median age was 53 (range 21- 79) at diagnosis, median tumour size was 17cm (12cm to 28cm) and median follow-up duration was 44.1 months. The most commonly encountered subtypes were de-differentiated liposarcoma (DDLPS) (56.6%), well-differentiated liposarcoma (WDLPS) (20.8%) and leiomyosarcoma (LMS) (11.3%) with a majority being high-grade (75.5%). The median disease-free interval was 2.9 years (2- 5.3 years) from the first surgery. The median age at second surgery was 56 (21- 79) and all patients had a complete resection (R0/R1). Recurrence patterns differed with subtypes where 90.9% and 9.1% of WDLS, 76.7% and 16.7% of DDLPS and 83.3% and 16.7% of LMS had local and distant relapses respectively from the second surgery. 62.5% of distant relapses was in the lung followed by nodes (18.8%) and liver (12.5%). The 5-year OS from the second surgery was 66.2% (95% CI: 54.3%- 80.8%). The 1-year, 3 years and 6 years DFS were 50.2% (95% CI: 38.2% - 65.9%), 10.4% (4.26% - 25.5%) and 3.91% (0.684% - 22.4%) respectively. Overall, 32 patients (60.4%) had passed away from sarcoma. The concordance indices for 6-year OS and DFS were 0.7 and 0.65 (Figure 1) respectively which represents a fairly accurate prediction by *Sarculator*.

**Conclusion:**

Our study has shown the *Sarculator* nomogram for primary recurrent was applicable in our cohort and its potential application in an Asian setting. The *Sarculator* nomogram will be a useful tool in clinical practice to improve risk stratification and facilitate prognosis-based decision-making. Moving forward, novel therapeutic strategies are required to enhance the prognosis of patients with recurrent RPS.

## Introduction

Retroperitoneal sarcomas (RPS) comprise up to 15% of soft tissue sarcomas (1). Retroperitoneal soft tissue sarcomas are divided into different subtypes with varying prognostic outcomes, including leiomyosarcoma, synovial sarcoma and liposarcoma, which are responsible for 75% of all cases (2, 3). The five-year overall survival rate for patients with RPS is up to 50% and reduces to 20% to 30% at ten years. (4) Several studies have shown that in 40% to 50% of patients, loco-regional recurrences occur within the first five years post-resection (5). While there are differences in recurrence rates between various histological subtypes, retroperitoneal liposarcoma tend to have a higher likelihood of recurrence locally with 20% of patients developing widespread metastases by five years post-resection (6). Notably, leiomyosarcoma (LMS), de-differentiated liposarcoma (DDLPS) and malignant peripheral nerve sheath tumours (MPNST) have a poorer prognosis compared to solitary fibrous tumour (SFT) and well-differentiated liposarcoma (WDLPS) (7). In addition, other factors, such as resection extent and tumour grade, contribute to patients’ prognostic outlook (8).

Notably, the American Joint Committee on Cancer (AJCC) staging system is limited in predicting patients’ prognosis with RPS where the TNM system is based on the anatomic stage of the tumour (9). It assumes the anatomic progression of the tumour correlates with tumour stage (10). As such, patients with the same anatomic progression are classified within the same tumour stage although prognosis could differ significantly due to the histological subtype. The AJCC has taken into account the need for customised prognostic tools that incorporate other tumour- and patient-related factors outside of the TNM staging system to achieve a more tailored and precise outcome prediction (11). Due to limitations of the AJCC staging system in predicting survival outcomes, nomograms have been developed that include key relevant factors in predicting survival probabilities (12, 13). To provide further prognostication, nomograms are useful tools in offering customised disease-specific estimates for patients with RPS (14, 15). A nomogram for first relapse locally recurrent RPS was developed using 602 patients from 22 centres. The recurrent RPS *Sarculator* is available in an electronic interface and includes variables of age, size, margins of re-resection, radiotherapy, chemotherapy and histology to predict for 6-year disease-free survival (DFS) and overall survival (OS) (16). Notably, it has not been validated externally. Hence, this study aims to validate the *Sarculator* recurrence nomogram in estimating the survival outcomes of patients with recurrent RPS in an Asian population as well as examine relapse patterns.

## Methods

All patients who had their first recurrent RPS from 1 January 2000 to 31 December 2017 with the first local relapse and eligible for curative re-resection were retrospectively analysed. Patients with incomplete diagnostic or treatment data were excluded from this study. In addition, patients with a histology of desmoplastic small round cell tumour, desmoid fibromatosis, Ewing, rhabdomyosarcoma and gastrointestinal stromal tumour (GIST) were excluded. This study was performed with approval of the Centralised Institutional Review Board of Singapore Health Services.

The type of surgery was unique for individual patients and suggestions of adjuvant therapy were based on globally recognised standards. The National Federation of Centres for the Fight Against Cancer) grading system was used. In addition, at least 2 qualified pathologists reviewed the histology at a multidisciplinary tumour board. Based on evidence-based guidelines, the extent of surgery was customised, and suggestions of adjuvant therapy were decided. All patients were reviewed at a follow-up visit 3 to 4 months post-operatively for the first 2 to 3 years and 6-monthly to a year after. A R0 or R1 margin is deemed as complete resection, including a microscopically negative margin (R0) and microscopically positive but macroscopically clear margin (R1). R2 is classified as an incomplete resection with tumour rupture or remaining disease. Harrell’s C concordance index was used to determine the nomogram’s discriminative ability and calibration plots to assess accuracy. For the calibration, the patients were divided into 3 groups. Death data was retrieved from the National Birth and Death registry for accuracy.

### Statistical Analyses

The various survival outcomes were assessed using Kaplan-Meier method. DFS was calculated from the date of primary surgery to local or distant metastases while OS was the date of primary surgery to death and censored at last follow up. Sarcoma-specific survival (SSS) was from the date of primary surgery to sarcoma-specific death and censored at the last follow-up. To determine significant predictors, univariate and multivariate analyses were performed.

Harrell C’s concordance index was used to assess discrimination (17). The index corresponds to the probability that the patient with the actual worse outcome will be deemed to have a higher chance of having the event first in a randomly selected pair of patients. Accordingly, perfect discrimination would be an index of 1 while random discrimination is a value of 0.5. If the concordance index was >0.7, this would suggest a good discriminative capability of the nomogram. All analyses were performed using R statistical software (Version 3.5.3).

According to the predicted survival probabilities, patients were stratified into three sub-groups for calibration. The level of calibration referred to the extent of concordance between predictions of the nomogram and actual outcomes of patients. This was assessed by having three groups of patients separated according to their nomogram-predicted probabilities. Subsequently, a plot was made of actual probabilities taken from the Kaplan–Meier estimate against the mean of the predicted probabilities for each group. Before generating the plots, calculation of the mean and 95% confidence interval of survival probabilities for each sub-group was performed.

## Results

There were a total of 53 patients included in this study. Patient and tumour characteristics have been summarised in **Table 1**. 66.0% had their first resection at the same centre. The median age was 53 (range 21- 79) at diagnosis, median tumour size was 17cm (12cm to 28cm) and median follow-up duration was 44.1 months. The majority of subtypes comprised of de-differentiated liposarcoma (DDLPS) (56.6%), well-differentiated liposarcoma (WDLPS) (20.8%) and leiomyosarcoma (LMS) (11.3%) with a majority being high-grade (75.5%). The median disease-free interval was 2.9 years (2- 5.3 years) from the first surgery.

**Table 1:** Patient and tumour characteristics.

Variable		n (%)
**Median age at diagnosis (Range) (n = 53)**		53 (21-79)
**Median size of primary tumour (IQR) (n = 53)**		17 [12-28]
**Histology (n = 53)**	DDLPS	30 (56.6%)
	LMS	6 (11.3%)
	MPNST	1 (1.9%)
	Others	3 (5.7%)
	SFT	2 (3.8%)
	WDLPS	11 (20.8%)
**Grade (n = 53)**	1	13 (24.5%)
	2	22 (41.5%)
	3	18 (34%)
**No of resected organs at first surgery (n = 53)**	0	20 (37.7%)
	1	21 (39.6%)
	2	9 (17%)
	3	2 (3.8%)
	4	1 (1.9%)
**Chemotherapy for primary tumour (n = 53)**	No	52 (98.1%)
	Yes	1 (1.9%)
**Radiotherapy for primary tumour (n = 53)**	No	46 (86.8%)
	Yes	7 (13.2%)
**Multifocality at second surgery (n = 53)**	No	27 (50.9%)
	Yes	26 (49.1%)
**Margins (n = 53)**	R0	13 (24.5%)
	R1	40 (75.5%)
**Median DFI from first surgery in year (IQR) (n = 53)**		2.9 (2 – 5)
**Local recurrence from second surgery (n = 53)**	No	10 (18.9%)
	Yes	43 (81.1%)
**Distant recurrence form second surgery (n=53)**	No	37 (69.8%)
	Yes	16 (30.2%)

IQR, inter-quartile range; DDLPS, de-differentiated liposarcoma; LMS, leiomysarcoma; MPNST, malignant peripheral nerve sheath fibrous tumour; SFT, solitary fibrous tumour; WDLPS, well-differentiated liposarcoma, DFI, disease-free interval.

**Figure 1:**
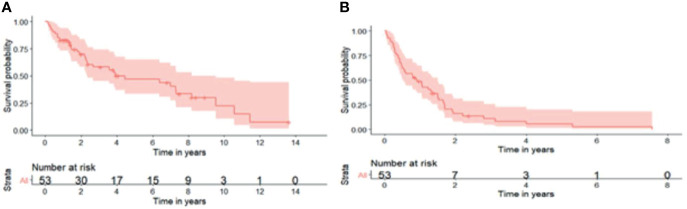
**(A)** 5-year overall survival from second surgery was 66.2% (95% CI: 54.3%- 80.8%). **(B)** 1-year, 3-years and 6-years disease-free survival were 50.2% (95% CI: 38.2%- 65.9%), 10.4% (95% CI: 4.26%- 25.5%) and 3.91% (95% CI: 0.684%- 22.4%) respectively.

All patients had their second resection at a single centre. The median age at second surgery was 56 (21- 79) and every patient had a complete resection (R0/R1) (**Table 2**). Patterns of recurrence differed with subtypes where 90.9% and 9.1% of WDLS, 76.7% and 16.7% of DDLPS and 83.3% and 16.7% of LMS had local and distant relapses respectively from the second surgery. 62.5% of distant relapses was in the lung followed by nodes (18.8%) and liver (12.5%). The 5-year OS from the second surgery was 66.2% (95% CI: 54.3%- 80.8%) (**Figure 1A**). The 1-year, 3 years and 6 years DFS were 50.2% (95% CI: 38.2% - 65.9%), 10.4% (4.26% - 25.5%) and 3.91% (0.684% - 22.4%) respectively (**Figure 1B**). Overall, 32 patients (60.4%) had passed away from sarcoma.

**Table 2:** Recurrent tumour characteristics.

Variable		n (%)
**Multifocality at second surgery (n = 53)**	No	27 (50.9%)
	Yes	26 (49.1%)
**Margins (n = 53)**	R0	13 (24.5%)
	R1	40 (75.5%)
**Median DFI from first surgery in year (IQR) (n = 53)**		2.9 (2 – 5)
**Local recurrence from second surgery (n = 53)**	No	10 (18.9%)
	Yes	43 (81.1%)
**Distant recurrence form second surgery (n = 53)**	No	37 (69.8%)
	Yes	16 (30.2%)

DFI, disease-free interval; IQR, inter-quartile range.

### Validation of Nomogram

**Figure 2:**
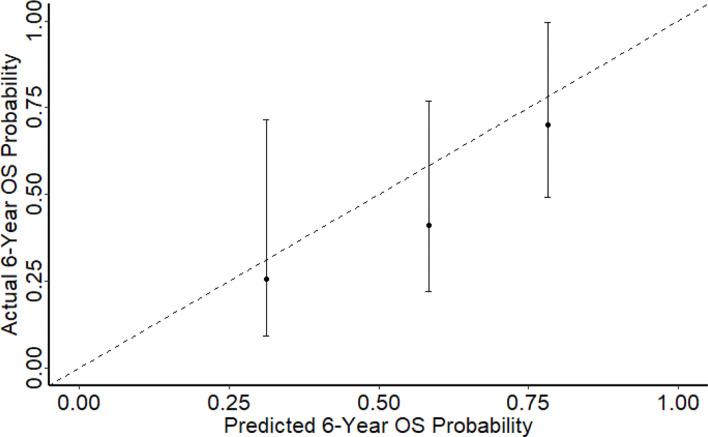
Calibration plot showing 6-year OS against predicted probabilities.

In comparison with the development set, our cohort of patients had similar median age, median tumour size, proportion of multi-focality and median follow-up. There was a larger proportion of patients diagnosed with DDLPS (46.8% *vs* 30.0%) and a lower radiotherapy rate (20.1% *vs* 36.9%). The concordance indices for 6-year OS and DFS were 0.70 and 0.65 (**Figure 2**) respectively which represents a fairly accurate prediction by *Sarculator*. The Harrel C statistic concordance indices obtained were similar to those from earlier validation studies of *Sarculator* (0.68-0.73 and 0.69-0.73 for OS and DFS).

## Discussion

In light of the limitation of the AJCC staging system in estimating the prognosis of RPS patients, nomograms are valuable clinical adjuncts in offering predicted survival outcomes (18). In particular, Gronchi et al. developed a nomogram for prediction of the 6-year OS and DFS based on locally recurrent RPS patients who underwent surgery for resection at 22 centres from 2002 to 2011 (16). The nomogram was developed based on age at second surgery, multifocality (multiple sites of tumour), grade, completeness of second surgery, histology, chemotherapy or radiotherapy at first surgery and number of organs resected at first surgery. It has been found to be accurate by internal validation. However, it has not been externally validated in an Asian population. External validation of the nomogram is essential to assess its applicability in different populations (19). Our study would be the first to incorporate the use of *Sarculator* in an Asian setting. Validation of the *Sarculator* recurrence nomogram was performed based on 53 patients in this study treated for recurrent RPS. Patient characteristics were largely similar between our cohort and the Italian patients in terms of tumour size, depth, histologic variant and location of tumour where in both institutions, the most common histology was liposarcoma. However, there was a difference in terms of the age of patients, with a higher median age of 64 compared to 56 in ours and developmental cohorts respectively. In terms of tumour size, histologic subtype, age at diagnosis, extent of resection and multi-focality, our cohort was similar to the developmental cohort used in *Sarculator*. Notably, the number of grade 1 tumour was lower in our cohort (19.3% *versus* 28.1%) and local recurrence rates were higher than Gronchi’s developmental set. 54 out of 107 patients (50.5%) had a recurrence locally while 23 (21.5%) patients had both local and distant recurrences. Nonetheless, it is difficult to compare both groups equally due to heterogeneity in patient cohorts.

This study used two approaches to validate the nomogram where the first was the extent of discrimination which was quantified using Harrell’s concordance index. The *Sarculator* recurrence nomogram achieved a high level of concordance at 0.70 in our study population. As such, the nomogram has a 70% chance of yielding a higher mortality probability if the patient with the shorter follow-up period died of locally recurrent RPS in a given pair of randomly selected patients. When applied to an external patient cohort, the nomogram predictions may be comparable with the actual observed overall survival, demonstrating that the nomogram reliably predicts outcome for patients with locally recurrent RPS. Notably, the c-index of 0.70 and 0.65 is similar to the 6-year predicted OS and DFS concordance index documented in the developmental cohort of 0.70 and 0.67 respectively. The c-index achieved in our study is also comparable to those reported in other external validation studies involving lung and gastric malignancies that ranged from 0.71 to 0.74 (20–21). Hence, the *Sarculator’s* concordance indices demonstrate that it has decent discriminative ability as the concordance index is higher than 0.7. The second approach used for nomogram validation was the level of calibration. Three calibrations plots were plotted for 6-year predicted OS probability. Notably, as the points on the plots were relatively aligned to the 45° line, this showed that the 6-year OS had good calibration. This demonstrates that the nomogram is universally accurate and applicable. Being the first study to externally validate the *Sarculator* recurrence nomogram, our findings support the integration of this nomogram into clinical practice. Nomograms are an accessible and reliable statistical method to predict survival outcomes (22). Furthermore, nomograms are better able to estimate survival outcomes compared to the conventional AJCC TNM staging for RPS, thereby facilitate decision‐making and adjuvant treatment (23). The *Sarculator* nomogram will be a useful tool in clinical practice to strengthen the prognosis-based decision making and enhance risk stratification. This tailored patient-centred approach will be particularly relevant as novel adjuvant therapies are being developed to improve the prognosis of recurrent RPS.

Our study also examined the local relapse patterns which varied with histological subtype. For example, patients with WDLPS had a higher incidence of local recurrences and sarcoma, while a significantly higher proportion of patients with LMS had distant recurrences. Recurrence patterns differed with subtypes where 90.9% and 9.1% of WDLS, 76.7% and 16.7% of DDLPS and 83.3% and 16.7% of LMS had local and distant relapses respectively from the second surgery. 62.5% of distant relapses was in the lung followed by nodes (18.8%) and liver (12.5%). These patterns of relapse have been reported in studies (24, 25). Similarly, in the developmental Italian cohort, recurrence patterns were specific to histological subtype with liposarcoma having the highest 6-year crude-cumulative incidence (CCI) of second local recurrence at 60.2% to 70.9% while LMS had a higher 6-year CCI of distant metastasis at 36.3% (16).

The strengths of our study lie in the similarity of our cohort to that of the developmental set with regards to age, percentage of patients with multi-focal tumours, tumour size and duration of follow-up. Notably, there were lesser patients with grade 1 tumours in our cohort. Furthermore, majority of patients in our cohort underwent surgery in a tertiary centre by the same surgeons. Moreover, death records were verified against the Singapore Registry of Births and Deaths. However, this study has a few limitations. These include the small sample size, retrospective nature as well as varying follow-up time points of our patients. Due to the retrospective nature and single-institution design of the study, there may be selection bias where some patients could have been excluded due to a short follow-up duration or important missing variables. Furthermore, patients referred from another institution who had a R2 resection were not included in this study. Moreover, the relatively small size of our cohort requires large external validation cohorts to substantiate our findings. Moreover, certain histology subtypes, including MPNST (n = 1) and SFT (n = 2), were under-represented in our cohort. Our cohort also comprised of patients from an Asian background and our findings may not be applicable in other populations. Nonetheless, this study is the first to examine the applicability of *Sarculator* in recurrent RPS in an Asian population. Therefore, the results from this study support and reinforce the urgent need for a future study with a larger patient population possibly in collaboration with other regional centres.

## Conclusion

Our study has shown the accuracy and potential usefulness of nomograms in an Asian population. The *Sarculator* recurrence nomogram for primary recurrent was applicable in our cohort and has a potential application in an Asian setting. The *Sarculator* recurrence nomogram will be a useful tool in clinical practice to support the decision-making process and enhance risk stratification. Moving forward, the application of this prognostic tool can help guide clinicians when counselling patients on survival probabilities, to generate surveillance recommendations in conjunction with identifying high-risk patients who may benefit from adjuvant treatment.

## Data Availability Statement

The original contributions presented in the study are included in the article, further inquiries can be directed to the corresponding author.

## Author Contributions

All authors contributed to data collection, analysis and formulation of manuscript. All authors contributed to the article and approved the submitted version.

## Funding

This study is supported by the NCCS Cancer Fund (Research). CAJO is supported by the National Research Council Transition Award (NMRC/TA/0061/2017). All the funding sources had no role in the study design, data interpretation or writing of the manuscript.

## Conflict of Interest

The authors declare that the research was conducted in the absence of any commercial or financial relationships that could be construed as a potential conflict of interest.

## Publisher’s Note

All claims expressed in this article are solely those of the authors and do not necessarily represent those of their affiliated organizations, or those of the publisher, the editors and the reviewers. Any product that may be evaluated in this article, or claim that may be made by its manufacturer, is not guaranteed or endorsed by the publisher.
